# Comparative analysis of ankyrin (ANK) genes of five capripoxviruses isolate strains from Xinjiang province in China

**DOI:** 10.1186/s12985-020-01407-w

**Published:** 2020-08-28

**Authors:** Chuanchuan He, Jianjun Tong, Xueping Zhang, Milikaimu Tuohetiniyazi, Yu Zhang, Youwen Li

**Affiliations:** 1 Key Laboratory of Tarim Livestock science Technology, Alar, 843300 Xinjiang China; 2 College of Animal Science in Tarim University, Alar, 843300 Xinjiang China; 3 College of Life Science in Tarim University, Alar, 843300 Xinjiang China

**Keywords:** Capripoxvirus, ANK genes, Specific signatures, Sequence analysis, Xinjiang of China

## Abstract

**Background:**

Sheeppox and goatpox are both economically important animal diseases in which pathogens are goatpox virus (GTPV) and sheeppox virus (SPPV). They can’t cause cross-species infection between sheep and goats in general. But in recent decades, the infection of sheep by goatpox or goats by sheeppox has been reported. The literature has indicated that the occurrence of these cases has a significant and direct relationship with mutations of ankyrin genes families (ANK genes 010,138,140,141.2,145) located in two-terminal regions of capripoxvirus genomes. So it is very important to decipher these nucleotides and their coding amino acid sequences of the five genes regarded as host range and virulence factors for effective prevention and control of capripoxvirus diseases.

**Methods:**

In this study, all the ankyrin genes of three goatpox virus, two sheeppox virus, and one GTPV vaccine strains from Nanjiang areas of Xinjiang province of China during 2010–2011 were collected, amplified, cloned and sequenced. The sequence of every ankyrin genes has been compared with not only sequences from six viruses but also all sequences from three species of capripoxvirus genus from Gene bank, and every ANK gene’s mutated nucleotides and amino acids have been screened, and the relationship of genetic evolution among different virus strains has been analyzed, as well as the domain architecture of these genes was forecasted and analyzed.

**Results:**

The six capripoxvirus strains can be well-distinguished GTPV and SPPV based on five ANK genes’ sequence identicalness except for GTPV-SS strain, which showed higher identicalness with SPPV. The ANK gene sequence of the GTPV-SS strain was 100% identical with SPPV-M1 (ANK138,140,145) and SPPV-M2 (ANK138,145), respectively. Phylogenetically, these six capripoxvirus strains were also grouped into the same cluster of India reference strains in lineages and showed extreme identical conservative or variable regions with India capripoxvirus isolates by sequence alignment. Moreover, for the functional domains, these ANK genes of capripoxvirus except for ANK gene 145, are identical in size, and ANK genes 145 of SPPV are usually 100 bp (approximately 30 aa) longer than those of GTPV and eventually form a PRANC domain at C-terminus.

**Conclusions:**

The isolated strain of GTPV-SS may be a cross-species infection or the collected material was contaminated, and the inferred Capripox outbreak in Xinjiang in 2010 can be introduced from India. ANK genes 138,140,141.2 and 145 of capripoxvirus can be used as the target genes to identify GTPV and SPPV. Moreover, the four ANK genes determining the host range are more significant than the ANK gene 010. These ANK genes play combining roles for their function.

## Background

Capripox is a typically contagious, epitheliotropic, fulminating disease, caused by capripoxvirus of genus Capripoxvirus of subfamily Chordopoxvirinae of family Poxviridae [27], which includes goatpox virus (GTPV), sheeppox virus (SPPV), lumpy skin disease virus (LSDV) of cattle. Goatpox and sheeppox are the most harmful ruminant animal diseases from goats and sheep in all pox diseases. Goatpox and sheeppox are mainly prevalent in central and southern Asia, central and northern Africa, and India sub-continent [9] and the part of the People’s republic of China (Tao [24, 28]), while LSD is as a local disease, mainly limited in Africa. Because of the high mobility, mortality and fatality of capripox causing a significant economic loss for stockbreeding every year [17], the disease has been listed as class A notifiable animal disease by the Office of International Epizootic (OIE) [7, 30]. Capripoxvirus originally has host specificity, i.e., sheeppox virus only infected sheep while goatpox virus only infected goat. However, in recent years, some isolates of capripoxvirus frequently show cross-infection between goats and sheep [5], while they can cause mild clinical symptoms in the non-corresponding host animal.

Both goatpox virus and sheeppox virus consist of 147 putative opening read frame (ORF). The length of full genomes is approximately 150 kb around, including central coding regions (ORF24–123) and two flanking regions (0RF1–23,124–156). Five putative ANK genes (ORF010,138,140,141.2,145) of capripoxvirus are located in the two flanking regions, respectively. ANK genes as extremely important a class of large superfamily genes, widely exist almost all of the organisms, from plants to animals and even human, or procaryote to eucaryote, playing an irreplaceable role in the evolution of life. ANK genes throughout the kingdom of the virus are mainly limited to poxvirus but not exclusively [12]. Generally, these ANK genes in function link the membrane-associated proteins [2], including transport and ion channels, acting on the effect of adhesion molecular, inhibiting viral-induced apoptosis [26] and et al. However, several official data and related literature have also indicated that the biological function of ANK genes family of capripoxvirus, as a host-range gene, has a significant effect on the host range of viral infection [11]. The characteristics of the ANK gene of capripoxvirus make it a potential candidate for the development of the capripox vaccine. Many live-attenuated vaccines of capripoxvirus present a common characteristic that ANK genes were disrupted with mutations [6]. Nevertheless, sporadically, this live-attenuated vaccine can cause diseases, and the vaccine fails to induce immunity [13]. Based on the modification of ANK genes, developing a single vaccine for all strains is the most effective way to prevent and control the capripoxvirus disease for cattle, sheep, and goats.

Hence, it is extremely important to figure out the underlying infection mechanism of capripoxvirus strains for developing an efficient vaccine. All the time, the ANK genes were most studied, but its exact role during infection is still unknown, especially for host range and virulence. To understand the infection mechanism of ANK genes is very more necessary. By analyzing these ANK genes sequences of capripoxvirus isolates and field strains and their lineages relationship based on the phylogenetic trees, an attenuated vaccine based on antigenic homology among all strains will be developed to protect cattle, sheep, and goats. The study aimed to provide some valuable data for developing a new generation vaccine against capripoxvirus.

## Materials and methods

### Virus

Five isolate strains of capripoxvirus were respectively collected from these tissues and scabs of goats or sheep infected capripox disease at different flocks of goats or sheep at Nanjiang areas of Xinjiang province, China during 2010–2011, with a GTPV vaccine strain obtained from Tecon biological pharmaceutical companies, China [31], which were saved at *Key Laboratory of Tarim Animal Husbandry Science* (Table 1)*.*

**Table 1:** Details of isolates of capripox used in the present study

Isolate/strain	Origin	Year of isolation	District	Accession
GTPV-Thx	goat	April .2010	Nanjiang	–
GTPV-KS	goat	April.2011	Nanjiang	–
GTPV-SS	goat	July.2011	Nanjiang	–
SPPV-M1	sheep	January.2011	Nanjiang	–
SPPV-M2	sheep	February.2011	Nanjiang	–
GTPV-YM	vaccine	–	–	–

### PCR and sequencing

According to the manufacturer’s instructions, viral genomes were extracted using a virus DNA extraction kit (Tiangen, Beijing, China); the specific primers were designed using Primer 5.0 software; PCR amplification was performed using T100™ Thermal PCR (Kezhida, Beijing, China). These primers for amplifying and sequencing were listed (Table 2); PCR products were checked by 1% agarose gel electrophoresis and purified by DNA Purification & Recovery Kit (Tiangen, Beijing, China). The target genes were cloned into the prokaryotic expression vector of pET32a (Youbia, Hunan, China) and transformed into *E.coli* DH5α. The selected positive clones were sent for sequencing by the biotech company of Sango (Shanghai, China).

**Table 2:** Universal primer of PCR amplification and sequencing of ANK genes of six capripoxvirus strains [10]

ORF	Primer sequence(5′-3′)	Length of amplicon (~bp)
ORF010	F1:CGGAATTCGCAATCATCAATTACTATGG (PCR) R1:CCGCTCGAGAACTTTTTATTGTTTGCC (PCR)	636
ORF138	F1:CGGAATTCGATGATTTTGTTATACGATTAC (PCR) R1:CCGCTCGAGTTATGGCGTATACACAAC (PCR) GTTATACGATTACATCACGCTC (Seq.) ATTCGTCAGGATCCAAAGTGAATAA (Seq.) TGGGATTTGTTGCGTATT (Seq.)	1896
ORF140	F1:CGGAATTCGTCACCAAAAATGTCACTGT (PCR) R1:CCGCTCGAGTTATAAAAAGTACTTCTTTG (PCR) TTGCTACTAAAAAACGGTGC (Seq.) TATCTAAGGATTTACGTGTATATGA (Seq.)	1497
ORF141.2	F1:CGGAATTCGACCATGGAATACATTAGTG (PCR) R1:CCGCTCGAGTCATTTTATTGCCAACAAC (PCR) ACAGTTTTGGGCAACAAAAG (Seq.)	1344
ORF145	F1:CGGAATTCGTATACTTACAAAATGGAT (PCR) R1:CCGCTCGAGTAAATTTTACTTTAACGG (PCR) AATACTTACAACGAGATTCGCC (Seq.)	1400

### Sequence analysis of ANK 010,138,140,141.2 and 145

Sequences are analyzed and aligned by Bioedit software using the Clustalw method [21]. Homology analysis of nucleotides and amino acids are respectively carried out by DNAman Version6.0 and DNAstar Version7.1.0 software. The sequencing results are repeatedly proofed by the peak map using the chromes software. Positions of the nucleotides and deduced amino acids of these ANK genes (010,138,140,141.2,145) are presented respectively using the GTPV India/P6 reference strain (Table 3). The domain architecture of these genes sequences is analyzed using the SMART (Simple Modular Architecture Research Tool) (http://smart.embl-heidelberg.de/) [19, 20].

**Table 3:** Accession number of the reference strains from the Gene bank database used in this study

Isolate	Accession number				
	010	138	140	141.2	145
GTPV AV41	MH381810.1	MH381810.1	MH381810.1	**/**	MH381810.1
GTPV India/P100	MF629128.1	MF629136.1	MF629110.1	MF629119.1	**/**
GTPV India/P62	MF629127.1	MF629135.1	MF629109.1	MF629118.1	MF629099.1
GTPV India/P6	MF629126.1	MF629134.1	MF629108.1	MF629117.1	MF629098.1
GTPV India/P21	MF629125.1	**/**	**/**	MF629116.1	**/**
GTPV India/P19	MF629124.1	MF629133.1	**/**	MF629115.1	MF629097.1
GTPV India/P3	MF629123.1	MF629132.1	**/**	MF629114.1	MF629096.1
GTPV FZ	KC951854.1	KC951854.1	KC951854.1	KC951854.1	KC951854.1
GTPV Gorgan	KX576657.1	KX576657.1	KX576657.1	KX576657.1	KX576657.1
GTPV G20-LKV	AY077836.1	AY077836.1	AY077836.1	AY077836.1	AY077836.1
GTPV Pellor	AY077835.1	AY077835.1	AY077835.1	AY077835.1	AY077835.1
LSDV Herbivac	MK441838.1	MK441838.1	MK441838.1	MK441838.1	MK441838.1
LSDV Cro2016	MG972412.1	MG972412.1	MG972412.1	MG972412.1	MG972412.1
LSDV LSD	KX764645.1	KX764645.1	KX764645.1	KX764645.1	KX764645.1
LSDV Herbivac	KX764644.1	KX764644.1	KX764644.1	KX764644.1	KX764644.1
LSDV Lumpyvax	KX764643.1	KX764643.1	KX764643.1	**/**	KX764643.1
LSDV Vaccine	AF409138.1	AF409138.1	AF409138.1	AF409138.1	AF409138.1
LSDV Saratov	MH646674.1	MH646674.1	MH646674.1	MH646674.1	MH646674.1
SPPV India/P42	MF629131.1	MF629138.1	MF629113.1	MF629122.1	**/**
SPPV India/P4	MF629129.1	**/**	MF629111.1	MF629120.1	**/**
SPPV Jaipur	MG000156.1	MG000156.1	MG000156.1	MG000156.1	MG000156.1
SPPV NISKHI	AY077834.1	AY077834.1	AY077834.1	AY077834.1	AY077834.1
SPPV A	AY077833.1	AY077833.1	AY077833.1	AY077833.1	AY077833.1
SPPV 10700–99	AY077832.1	AY077832.1	AY077832.1	AY077832.1	AY077832.1
SPPV India/P7	MF629130.1	MF629137.1	MF629112.1	MF629121.1	**/**
LSDV 155920	KX894508.1	KX894508.1	KX894508.1	KX894508.1	KX894508.1
LSDV Dagestan	MH893760.2	MH893760.2	MH893760.2	MH893760.2	MH893760.2
LSDV GR/15	KY829023.3	KY829023.3	KY829023.3	KY829023.3	KY829023.3
LSDV Bujanovac	KY702007.1	KY702007.1	KY702007.1	KY702007.1	KY702007.1
LSDV 0240	KX683219.1	KX683219.1	KX683219.1	KX683219.1	KX683219.1
LSDV 2490	AF325528.1	AF325528.1	AF325528.1	AF325528.1	AF325528.1
LSDV Warmbaths	AF409137.1	AF409137.1	AF409137.1	AF409137.1	AF409137.1
GTPV Turkey	MN072622.1	MN072622.1	MN072622.1	MN072622.1	MN072622.1
GTPV India	MN072620.1	MN072620.1	MN072620.1	MN072620.1	MN072620.1
GTPV Vietna	MN072621.1	MN072621.1	MN072621.1	MN072621.1	MN072621.1
GTPV Yemen	MN072625.1	MN072625.1	MN072625.1	MN072625.1	MN072625.1
GTPV Sudan	MN072624.1	MN072624.1	MN072624.1	MN072624.1	MN072624.1
SPPV Turkey	MN072631.1	MN072631.1	MN072631.1	MN072631.1	MN072631.1
SPPV Saudi	MN072630.1	MN072630.1	MN072630.1	MN072630.1	MN072630.1
SPPV Pendik	MN072629.1	MN072629.1	MN072629.1	MN072629.1	MN072629.1
SPPV Saudi	MN072627.1	MN072627.1	MN072627.1	MN072627.1	MN072627.1
SPPV Abu	MN072626.1	MN072626.1	MN072626.1	MN072626.1	MN072626.1
GTPV Oman	MN072623.1	MN072623.1	MN072623.1	MN072623.1	MN072623.1
SPPV Nigeria	MN072628.1	MN072628.1	MN072628.1	MN072628.1	MN072628.1
LSDV Kenya	MN072619.1	MN072619.1	MN072619.1	MN072619.1	MN072619.1
SPPV Fenner	**/**	MG000157.1	MG000157.1	MG000157.1	**/**

### Analysis of phylogenetic tree based ANK010,138,140,141.2 and 145

Phylogenetic trees of these ANKs genes sequenced in the current experiment, as well as the reference strains (Table 3), retrieved from Gene bank database, were respectively constructed carrying out the ‘Kimura 2-parameter model’s subroutine of the statistical method of Neighbor-Joining Tree by Mega v6.0 software [23].

## Result

### Sequence analysis of ANK genes010,138,140,141.2,145

The result showed that some ANK genes present frameshift mutation and can’t normally or completely encode amino acids because of inserting or deleting of nucleotides, i.e., ANK gene138 (insert A in position 142) of KS strain, ANK gene141.2 (insert A in position 724) of M1 strain, ANK gene140 (deletion A, A in position 28,37 respectively) of M2 strain, ANK gene138 (insert A in position 142) and gene140 (insert C, deletion T in position 1451,1460.respectively) and 141.2 (deletion T,T in position 790,806 respectively) of YM vaccine strain. Also, there were the 100% identical nucleotide sequences in different virus strains, like ANK gene010 (KS and M1 strains), ANK gene138 (SS, M1 and M2 strains or KS and YM strains), ANK gene140 (SS and M1 strains), ANK gene141.2 (Thx and KS strains), ANK gene145 (SS, M1 and M2 strains), and ANK genes (010,138,140,141.2,145) of the six Xinjiang strains and GTPV India/P62 reference strain were respectively up to 97.18–100%,96.22–99.74%,96.33–99.93%,96.33–99.93%,95.67–99.78% nucleotide similarity, and 96.2–100%,96.2%,96.2–100%,93.8–99.8%,92.7–99.6% amino acids similarity (Table 4).

**Table 4:** Similarity analysis of these Xinjiang-strains of capripox and the reference strain GTPV India/62 retrieved from Gene Bank

Isolate	Identity%(nucleotide/amino acid)				
	GTPV India/P62				
	ORF010	ORF138	ORF140	ORF141.2	ORF145
KS	100.00/100.0	99.74/−	99.93/100.0	99.93/99.8	98.60/97.8
Thx	99.69/99.1	99.79/99.8	99.73/99.6	99.93/99.8	99.78/99.6
YM	99.53/99.5	99.74/−	99.33/−	99.33/−	99.34/97.8
SS	97.18/96.2	96.22/96.2	96.79/96.2	96.73/94.0	95.67/92.7
M1	100.00/100.0	96.22/96.2	96.79/96.2	96.36/−	95.67/92.7
M2	97.80/96.2	96.22/96.2	96.33/−	96.65/93.8	95.67/92.7

**Fig. 1:**
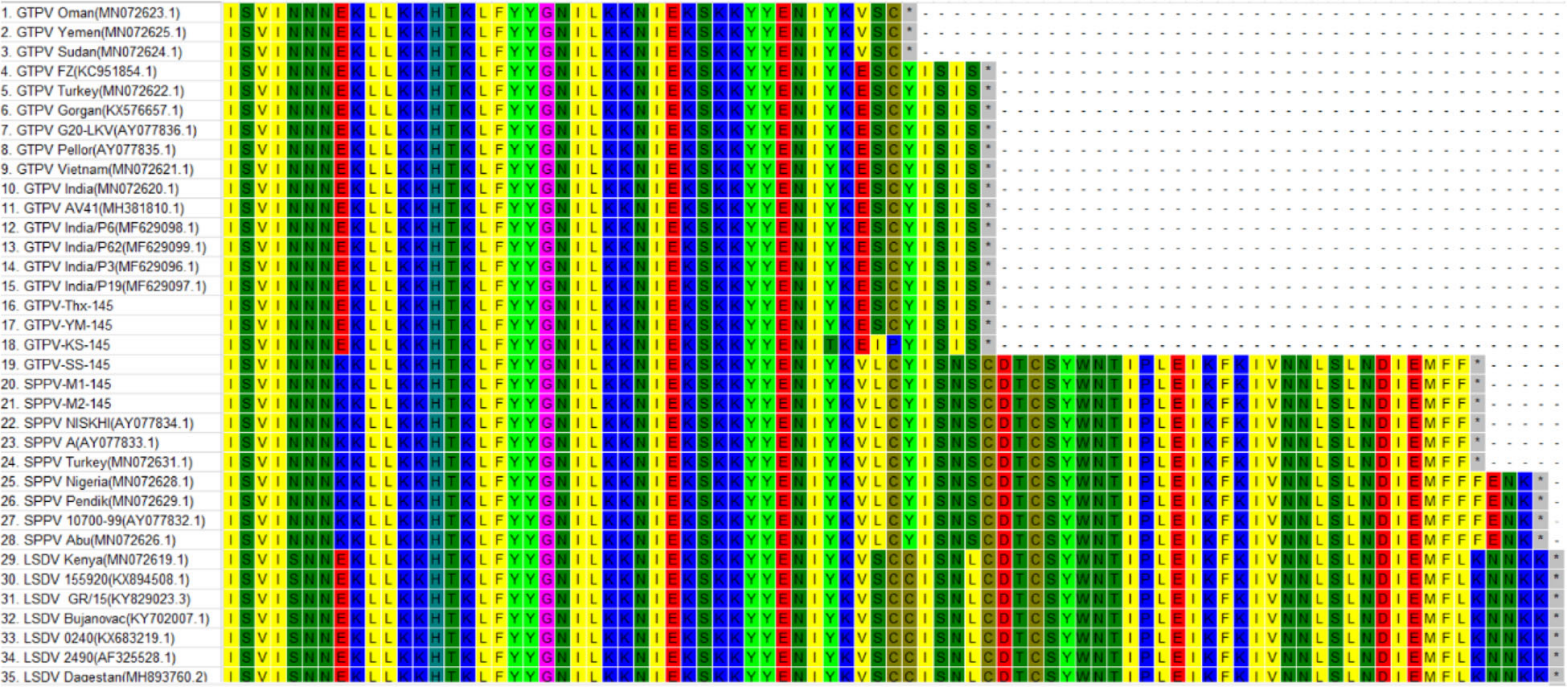
Difference analysis of coding amino acid of ANK gene 145 from three lineages (GTPV, SPPV, LSDV) of capripoxvirus strain

Based on these ANK genes (010,138,140,141.2,145), some specific signatures to distinguish the goatpox virus and sheeppox virus were demonstrated. ANK gene138 has 61 nucleotide positions changed, which result in 25 amino acid positions differentia between goatpox virus and sheeppox virus (Table 5); the ANK gene140 has 44 nucleotide positions changed, which result in 17 amino acid positions differentia between goatpox virus and sheeppox virus (Table 6); the ANK gene 141.2 has 40 nucleotide positions changed, which result in 23 amino acid positions differentia between goatpox virus and sheeppox virus (Table 7); ANK gene145 has 58 nucleotide positions changed, which result in 29 amino acid positions differentia between goatpox virus and sheeppox virus (Table 8). Besides, the comparative analysis found that another an obvious contrast of GTPV and SPPV focused on ANK 145 after respective comparing the five ANK genes (010,138,140,141.2 and 145) and coding amino acids of GTPV were shorter 30 around than those of SPPV and LSDV, and in lineages, SPPV had the closer relationship with LSDV (Fig.1).

**Table 5:** Unique nucleotide/amino acid signatures observed in ANK gene 138

SL.No	Nucleotide position and difference				Amino acid position and difference			
	Position	GTPV	SPPV	LSDV	Position	GTPV	SPPV	LSDV
1	24	A	C	T				
2	30	T	C	T				
3	90	A	G	A				
4	102	T	C	T/C				
5	135	C	T	C				
6	309	G	A	G				
7	338	T	C	C	113	L	S	S
8	360	A	G	A				
9	361	A	G	A/G	121	S	G	S/G
10	387	C	T	C				
11	513	C	T	C/T				
12	547	C	T	T	183	L	F	F
13	555	T	C	C				
14	564	G	A	G				
15	567	G	A	A/G				
16	614	G	A	A	205	S	N	N/D
17	629	C	T	C	210	T	I	T/S
18	648	T	C	C				
19	651	A	C	C				
20	675	C	T	C/T				
21	699	A	G	A				
22	795	A	G	A				
23	920	T	C	C	307	L	S	S
24	928–929	GT	AC	AC	310	V	T	T
25	980	G	A	A	327	R	K	K
26	1016	A	G	A	339	N	S	N
27	1044	T	G	G/A				
28	1089	G	A	G	363	M	I	R
29	1102	G	T	G	368	A	S	A
30	1137	T	C	C				
31	1292–1293	GT	AC	GT	431	S	N	S
32	1308	A	G	T/A				
33	1425	T	A	T				
34	1428	T	C	C/T				
35	1454	G	A	A	485	R	K	K
36	1482	G	A	A				
37	1535	C	T	C/T	511	S	L	S/L
38	1542	C	T	C/T				
39	1543	T	G	A				
40	1545	T	A	A	515	Y	E	K
41	1551	T	C	C/T				
42	1589	A	G	G	530	N	S	S
43	1602	G	A	G				
44	1606	G	A	G	536	V	I	V
45	1609	T	C	T	537	S	P	S
46	1617	T	C	C				
47	1618	G	A	G	540	D	N	D
48	1662	A	G	A				
49	1671	G	A	G	557	M	I	M
50	1678	A	G	A				
51	1679	T	G	G	560	I	G	R
52	1692	A	G	A				
53	1706	C	G	C	569	A	G	A
54	1764	T	C	C				
55	1770	C	T	C				
56	1854	G	A	G				
57	1875	C	A	C				
58	1888	G	T	G	630	V	F	V
59	1896	T	A	T	631	Y	–	Y
60	1897–1905	AAGCCATAA	–	AAGCCATAA	633–634	KP	–	KP

**Table 6:** Unique nucleotide/amino acid signatures observed in ANK gene 140

SL.No	Nucleotide position and difference				Amino acid position and difference			
	Position	GTPV	SPPV	LSDV	Position	GTPV	SPPV	LSDV
1	21	C	T	C				
2	24	T	C	C				
3	49	A	G	G	17	N	D	D
4	105	T	C	C/T				
5	147	G	T	G				
6	156	T	C	T				
7	162	T	G	G				
8	164	G	A	G	55	R	K	R
9	175	G	A	G	59	E	K	E
10	186	C	T	T				
11	241	T	C	T	81	Y	H	Y
12	280	C	T	C				
13	300	A	T	G				
14	301	A	G	G	101	N	D	D
15	342	T	G	G	114	I	M	M
16	387	G	A	G				
17	408	A	T	A				
18	438	G	A	A/G				
19	453	C	T	T				
20	472	C	T	T	158	L	F	F
21	492	C	T	T				
22	501	C	T	T				
23	552	G	T	G				
24	571	G	A	G	191	D	N	D
25	669	A	G	G				
26	679	T	C	C				
27	690	C	T	C				
28	748	A	T	T	250	I	L	L
29	763	G	A	G	255	E	K	E
30	873	T	C	T				
31	876	T	A	A				
32	885	A	C	C				
33	931	G	T	G	311	A	S	A
34	937	A	G	G	313	K	E	E
35	942	A	C	C				
36	1019	G	A	G	340	S	N	S
37	1077	A	G	G				
38	1170	G	T	G	390	K	N	K
39	1218	A	G	A				
40	1309	G	A	A	437	D	N	N
41	1322	G	A	G	441	R	K	R
42	1359	T	G	G				
43	1366–1368	ATA	TAT	TAT	456	I	Y	Y
44	1417	C	T	C	473	H	Y	H

**Table 7:** Unique nucleotide/amino acid signatures observed in ANK gene 141.2

SL.No	Nucleotide position and difference				Amino acid position and difference			
	Position	GTPV	SPPV	LSDV	Position	GTPV	SPPV	LSDV
1	16	G	A	G	6	D	N	D
2	24	A	C	A	8	E	D	E
3	25	G	A	G	9	E	K	E
4	39	G	A	G				
5	107	C	A	C	36	T	K	T
6	161	T	G	G	54	I	S	S
7	338	C	A	C	113	T	K	T
8	355	A	G	G				
9	357	C	T	T	119	N	D	D
10	376	T	C	T/C				
11	402	T	G	G	134	N	K	K
12	442	A	G	G	148	N	D	D
13	507	T	G	G/T	169	D	E	E/D
14	552	T	C	C				
15	654	C	T	T				
16	684	A	C	C				
17	708	A	G	A				
18	714	A	T	A				
19	717	A	G	A				
20	756	T	C	T				
21	758	G	A	G	253	S	N	S
22	789	T	A	T/C				
23	827	C	T	C	276	T	I	T
24	870	C	A	C				
25	887	G	A	G	296	C	Y	C
26	896	G	A	A	299	S	N	N/D
27	899	A	T	A	300	Y	F	Y
28	907	G	A	G	303	D	N	D
29	958	G	A	G	320	E	K	E
30	997	A	G	G	333	K	E	E
31	1005	C	T	C				
32	1063–1064	AA	GG	AG	355	N	G	S
33	1078	G	A	G	360	D	N	D
34	1121	G	T	G	374	R	M	R
35	1124	A	G	A	375	Y	C	Y
36	1211	A	G	G	404	K	R	R
37	1230	T	G	G	410	N	K	K
38	1246	G	A	A	416	D	N	N
39	1252-	T	G	G/A				
40	1253	T	C	C	418	L	A	A/T
41	1296	C	A	C				
42	1308	A	G	G				
43	1334	C	T	C	445	A	V	A

**Table 8:** Unique nucleotide/amino acid signatures observed in ANK gene 145

SL.No	Nucleotide position and difference				Amino acid position and difference			
	Position	GTPV	SPPV	LSDV	Position	GTPV	SPPV	LSDV
1	32	C	T	C	11	A	V	A
2	39	G	T	G	13	R	S	R
3	40	G	A	G	14	G	S	G
4	47	A	C	C	16	H	P	P
5	72	C	T	T				
6	73	A	G	G	25	N	D	D
7	96	A	G	G				
8	99	C	T	T				
9	141	C	T	C				
10	166	C	T	T				
11	204	G	A	A				
12	219	A	G	A				
13	276	T	C	C				
14	304	A	G	A	102	N	D	N
15	310	G	A	G	104	D	N	D
16	321	G	A	A				
17	325	C	T	T				
18	388	C	A	C	130	Q	K	Q
19	391	G	A	A	131	V	M	M
20	399	A	C	C				
21	400	G	A	G	134	E	K	E
22	412	C	T	C				
23	466	G	A	A	156	V	I	I
24	474	C	T	C/T				
25	570	T	C	T				
26	581	G	A	G	194	R	K	R
27	600	G	A	G				
28	645	C	A	A	215	N	K	K
29	690	C	T	C				
30	691	A	G	G	231	N	D	D/E
31	720	T	C	C				
32	733	A	G	A	245	T	A	T
33	742	A	G	A	248	N	D	N
34	768	A	G	A				
35	841	A	G	A	281	N	D	N
36	867	A	C	C				
37	868	A	G	G	290	N	D	D
38	898	A	C	C	300	S	R	R
39	902	A	G	G	301	N	S	S
40	917	C	A	A	306	T	N	N
41	960	C	T	T				
42	967	T	A	T	323	Y	N	Y
43	973	A	C	C	325	I	L	L
44	985	C	A	C				
45	999	C	T	C				
46	1015	A	G	A	339	I	V	I
47	1048	C	T	C	350	H	Y	H
48	1074	C	T	C				
49	1077	T	C	C				
50	1081	C	A	C				
51	1088	G	T	G	363	R	L	R
52	1194	C	T	C/T				
53	1224	G	T	G				
54	1237	G	A	G				
55	1281	C	T	T				
56	1337	A	T	T	446	E	V	V
57	1355	T	A	A	452	I	N	N
58	1362	A	T	C/T	454	–	C	C

### Analysis of phylogenetic trees

**Fig. 2:**
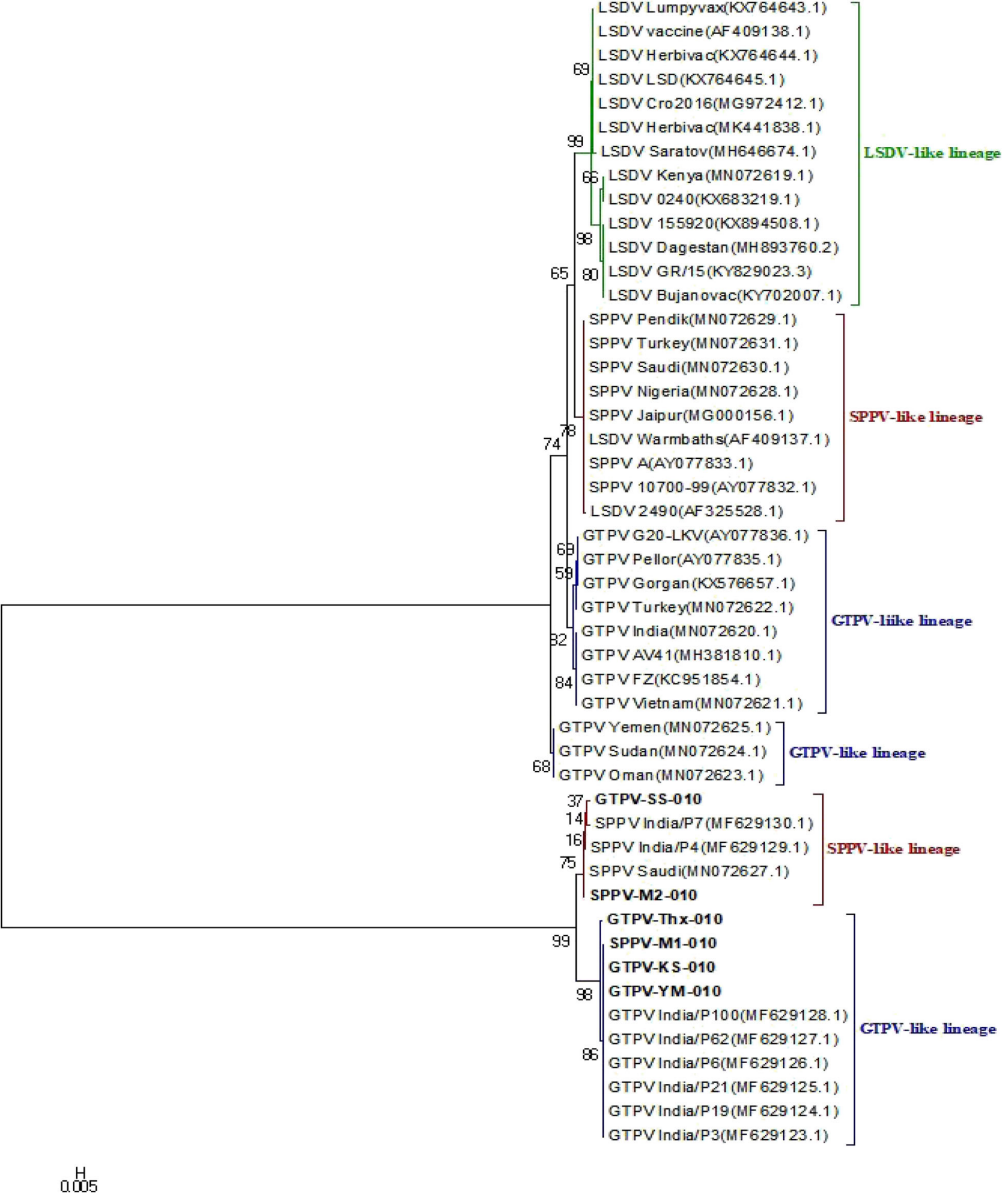
The phylogenetic trees of capripoxvirus based on ANK gene 010. The phylogenetic tree was constructed by the neighbor-joining method using MEGA V6.0 software

**Fig. 3:**
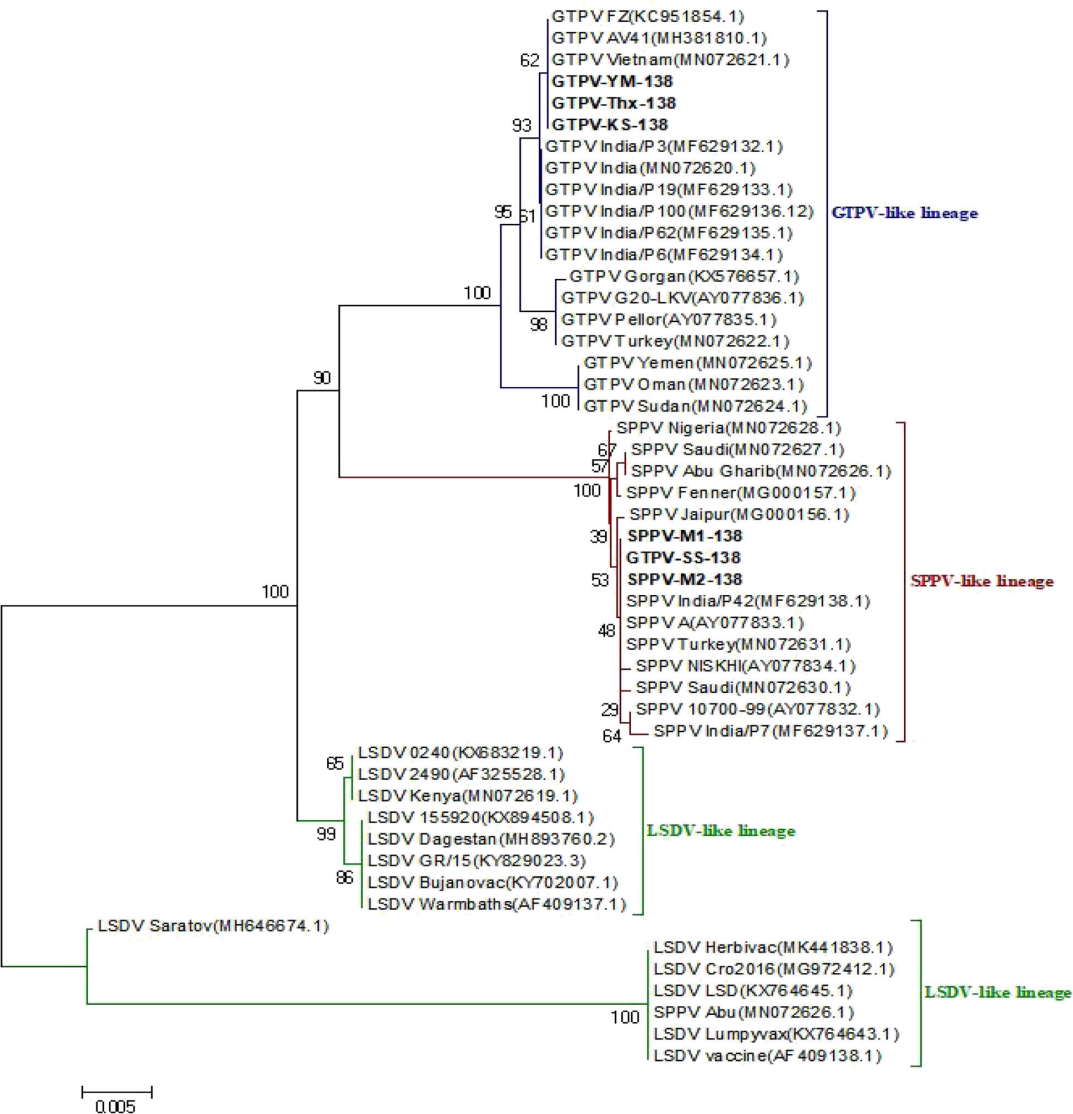
The phylogenetic trees of capripoxvirus based on ANK gene 138. The plylogenetic tree was constructed by the neighbor-joining method using MEGA V6.0 software

**Fig. 4:**
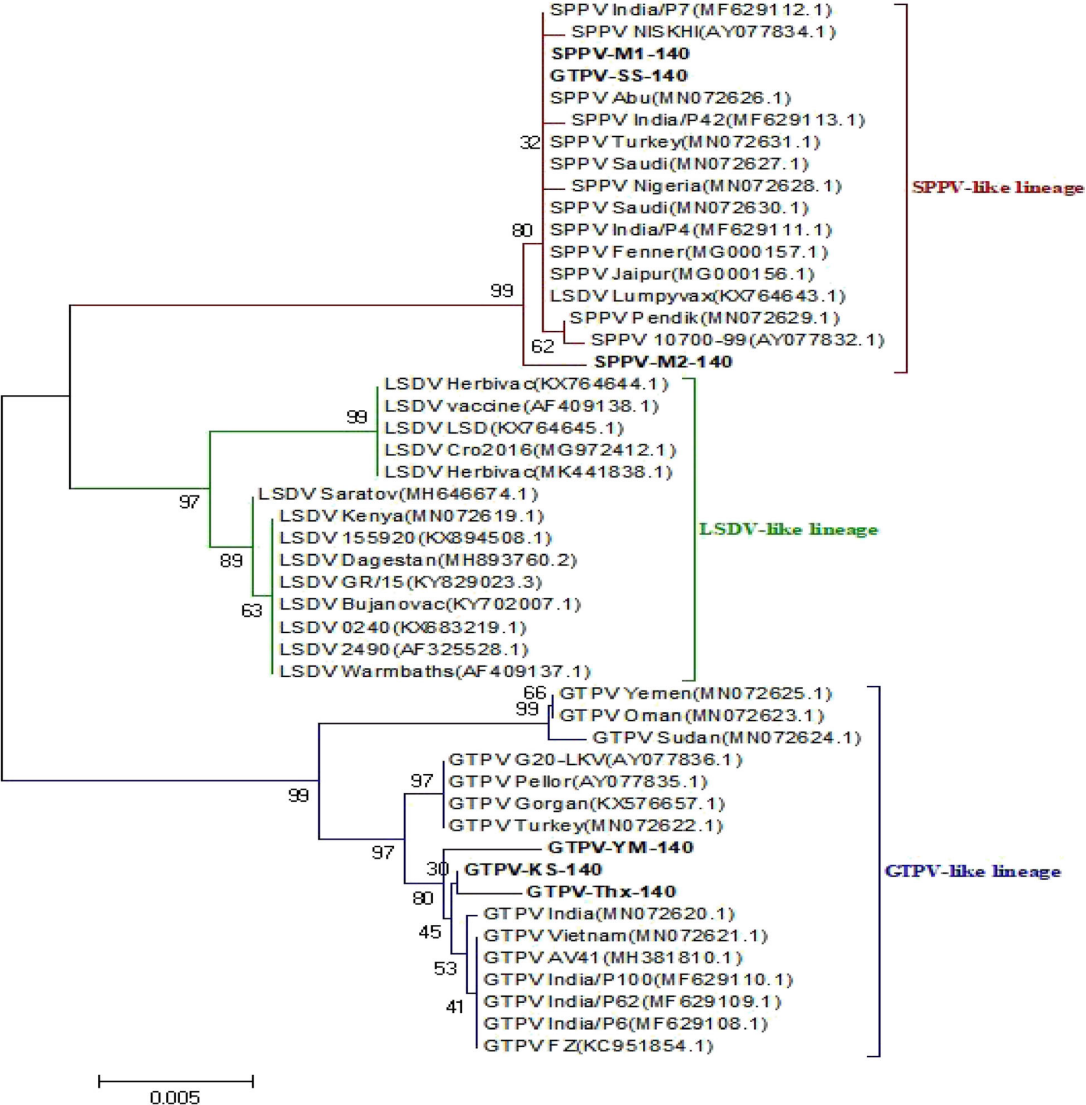
The phylogenetic trees of capripoxvirus based on ANK gene 140. The phylogenetic tree was constructed by the neighbor-joining method using MEGA V6.0 software

**Fig. 5:**
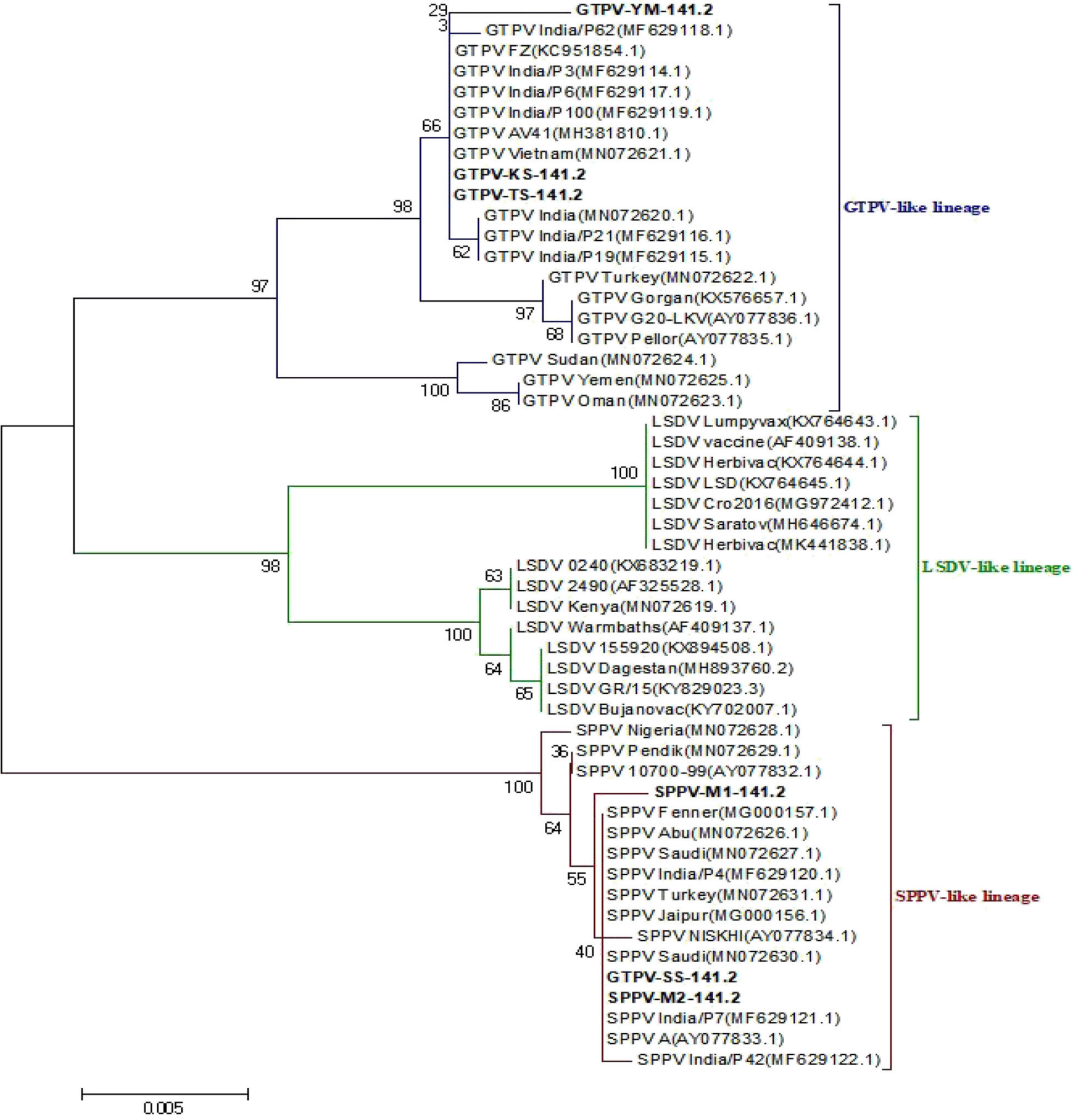
The phylogenetic tree of capripoxvirus based on ANK gene 141.2. The plylogenetic tree was constructed by the neighbor-joining method using MEGA V6.0 software

**Fig. 6:**
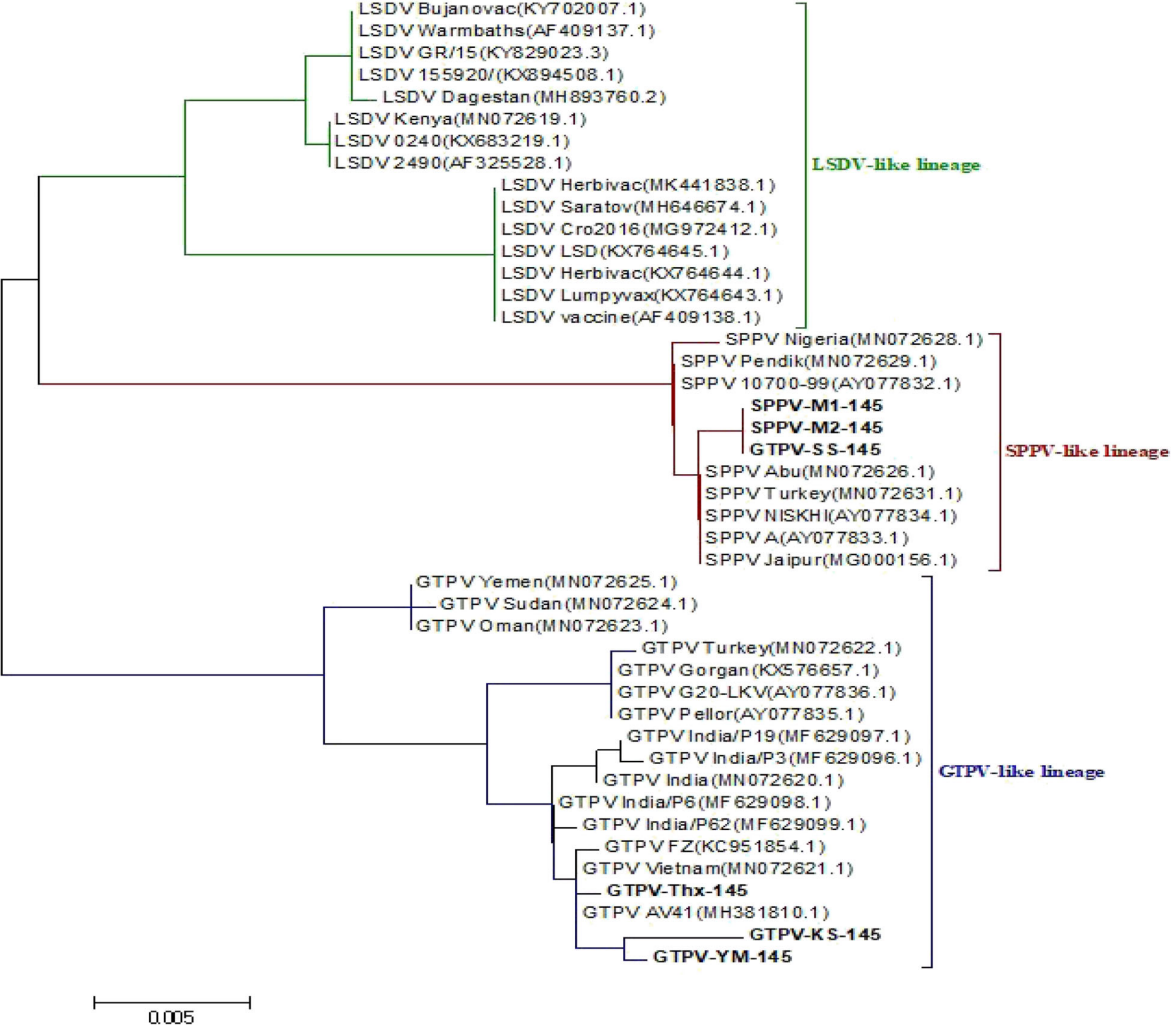
The phylogenetic tree of capripoxvirus based on ANK gene 145. The plylogenetic tree was constructed by the neighbor-joining method using MEGA V6.0 software

Based on the phylogenetic trees, 5 ANK genes (010,138,140,141.2,145) of 3 GTPV, 2 SPPV, and 1 GTPV vaccine strains were respectively constructed using Mega 6.0 software. Phylogenetically, these genes were grouped the same clusters or sub-clusters of India, Vietnam, Turkey, and Abu reference strains retrieved from the Gene bank database but shown the closest relationship with India reference strains from a genetic distance. ANK gene 010 was grouped into the cluster mostly comprised of India capripoxvirus strains (Fig. 2). So that the source of these capripoxvirus strains isolated in Xinjiang, we concluded that could be India. Also, the six capripoxviruses were classified into two sections from the relationship (Figs. 3, 4, 5 and 6). SPPV-M1, SPPV-M2, and GTPV-SS strains were much closer to the SPPV lineages, which GTPV-Thx, GTPV-KS, and GTPV-YM were much closer with the GTPV lineages. Interestingly, five ANK genes of GTPV-SS strain were contrarily classified as the SPPV lineages, and we inferred that the SS strain itself could be, in fact, a sheeppox virus strain.

**Fig. 7:**
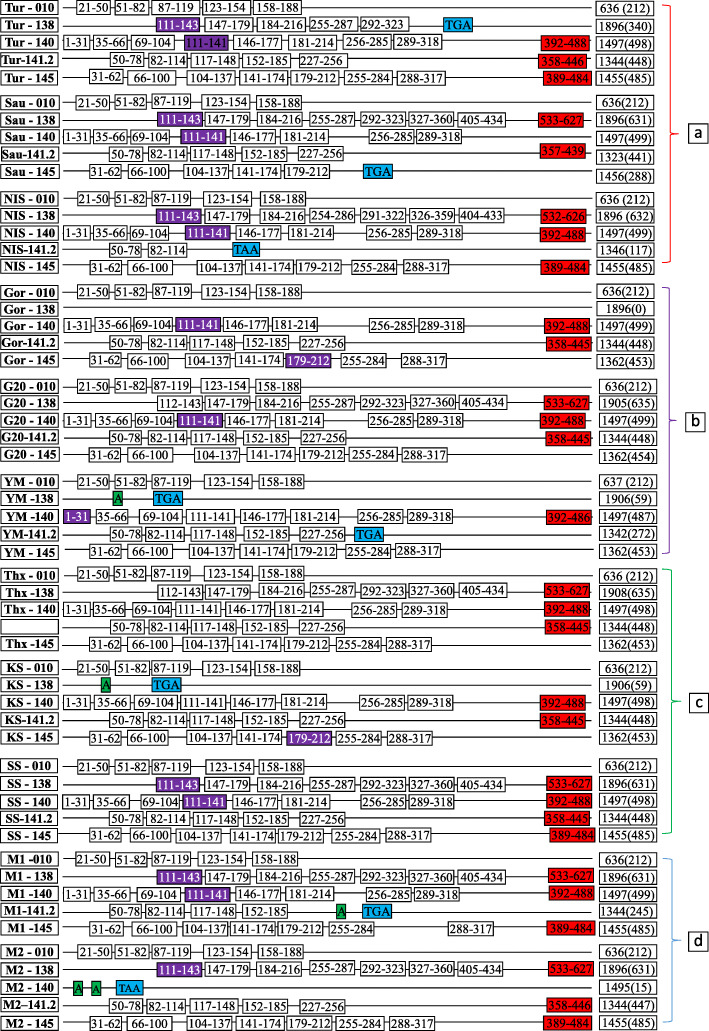
Analysis of domain architecture of ANK genes of capripoxvirus. These letters in the right, such as “ a, b, c and d” respectively represent SPPV vaccines (Accession numbers: *MN072631.1, MN072627.1* and *AY077834*), GTPV vaccine (Accession numbers: *KX576657.1* and *AY077836*), GTPV field strain and SPPV field strains. ANK repeats domains of capripoxvirus were, by one by, marked in white/purple frames, and color contrast representative different superfamily of ANK genes. The PRANC domains were marked in red frames. There are two numbers in the right frame; the former letter represents the nucleotides of these genes, and the later represents the number of encoding amino acids for the genes

#### Analysis of domains architecture of ANK genes of capripoxvirus

Five isolate strains and GTPV vaccines of capripoxvirus used to the current experiment and five attenuating vaccines reference strains of capripoxvirus collected from Gene bank database, were contrasted and analyzed according to SMART (Simple Modular Architecture Research Tool) (http://smart.embl-heidelberg.de/) (Fig. 7). From the analysis results, it was shown that the domains architecture of these ANK genes of capripoxvirus consist of several ANK repeats motifs at N-terminus and PRANC domains at C-terminus. For GTPV and SPPV, their ANK genes have the same numbers’ ANK repeats domains and PRANC domains and in contrast the difference that in the terminus domains of ANK genes 145 of SPPV there is also a PRANC domain but no in GTPV. Further analyzing field strains and vaccine strains of capripoxvirus found that frameshift is especially common in vaccines than in field strains. Besides, whether field strain or vaccine strains, their ANK genes 010 can always encode normally, and frameshift were also present in other ANK genes.

## Discussion

Capripox is the most serious animal poxvirus disease by far, and the infection of the host species named the three viruses (goatpox virus, sheeppox virus, and lumpy skin disease viruses). Hence, the diseases they caused clinical symptoms are so similar that it is difficult to distinguish by clinical symptoms or serology [3], but its pathogen kinds are still easily diagnosed by the host range. In theory, the three capripoxviruses have strict host-specificity. However, in recent years, the cases that SPPV infected goats or GTPV infected sheep, even these pathogens infected humans [1, 29], were increasing. In this study, we named an isolated strain as GTPV-SS as it comes from goats, but it was supposed to be SPPV as its gene sequences showed higher homology with SPPV than GTPV. There is no doubt that the diagnose of pathogen-species gets more difficult. If there is a crossing infection, and the pathogen identification can only be determined by PCR sequencing [26]. However, the genome’s homology of three viruses is more than 96%, and their many genes may be very similar and cannot be identified by PCR [32]. Many data and published literature have shown that the five ANK genes of capripoxvirus were closely related to the host of viral infection so that they may be a target of well-differentiated three capripoxviruses. The current experiment amplified all ANK genes from three GTPV strains, two SPPV strains, and a GTPV vaccine strain and sequencing analysis, to determine the capripoxvirus’ marker gene of species identification and research them as host range genetic mechanism.

Via sequence alignment and analysis, among five ANK genes of each isolates strains of capripoxvirus have the extremely high similarity, up to 99%, consistent with the result that the intact genomes similarity of capripoxvirus were up to 96% [25, 32]. ANK gene 145 of SPPV was obvious 100 bp longer than those in GTPV (Fig. 1); another four ANK genes show no interspecies difference. Further analyzing the unique nucleotides/amino acid signatures of the interspecies difference for each ANK genes from three species of capripoxvirus, each of ANK genes have different specific nucleotide/amino acid positions, but at the same position of these characteristic sites, at least two of three species were identical, all three species of capripoxvirus with a difference is uncommon, which explained that the source of GTPV and SPPV might evolve from the LSDV missing 9 ORFs [6, 26], eventually evolving three species in the process of constant generation [26]. The distribution of characteristic positions of ANK genes of five isolate strains from poxvirus in Xinjiang showed a slight difference with the previous result. In these ANK genes 138,140,141.2 and 145 of capripoxvirus in Xinjiang, we found some specific signature position to distinguish GTPV and SPPV. Ashwini et al. analyzed these different capripoxvirus strains in India and found that the four ANK genes (010,138,140, and 141.2) of capripoxvirus could be used as the target genes to distinguish GTPV and SPPV [10]. In contrast, these capripoxviruses are not only from Xinjiang, China but also from India, their ANK genes 138,140 and 141.2 have almost completely identical specific signatures, while these specific signatures of ANK 010 from capripoxvirus in India are found in the across-species in Xinjiang isolate strains of capripoxvirus, further reveled that ANK genes 138,140 and 141.2 are more conserved than ANK 010, more suitable to be used as the target genes to distinguish GTPV and SPPV.

Phylogenetically, the five ANK genes of Xinjiang strains of capripoxvirus were main grouped the same clusters or sub-cluster of India reference strains from the Gene Bank database. The inferred that the capripox outbreak in 2010 in Xinjiang could be introduced from India. Via further analysis, the five ANK genes of GTPV-SS strain were all grouped in SPPV lineages, and this result is consistent with the nucleotide analysis result, which ANK genes of GTPV-SS were 100% identical with SPPV-M1 (ANK gene138,140,145) and SPPV-M2 (ANK gene138,145). It is indicated that even if the source of GTPV-SS was originally from goats, but the isolate was an SPPV strain and can be a typical case of SPPV infecting goats, further indicating phylogenetic analysis in the class identification of a pathogen is a fast and scientific way.

Ankyrin (ANK) genes, as a class of significant superfamily genes, broadly distributed in nature [16]. ANK genes, in general, contains disparate ANK repeat motifs at -N terminus, and an F-box like/PRANC domains at C-terminus [8, 18] but in contrast, the domains at C-terminus exist divergent [4]. The PRANC domains was an F-box like domains [15], annotated in Pafm database, while can be a mutant via original F-box motif truncated [11], and in the function, the PRANC domains of poxvirus were closely related with F-box family and had similar roles. The previous study indicated that ANK repeats correlate with the host of viral infecting, while F-box domains mediated the interaction with the cellular SCF1 ubiquitin ligase [22]. Capripoxvirus genomes only contain five ANK genes. In this study, we sequenced and analyzed the function domain of these ANK gene sequences from five capripoxvirus isolates strains in Xinjiang and some vaccine strains from Genbank.

From the domains architecture of ANK genes, the majority of these genes from five ANK genes of capripoxvirus have the disparate ANK repeat domains, and some ANK genes contain an F-box like domains at C-terminus. The same gene from different virus strains still has larger differences. Analysis of these data found that the functional domains of ANK genes 010 from whether isolate strains or vaccine strains, or SPPV strains or GTPV strains, are more stable, and all contain five ANK repeat motifs, but no the F-box like domains. The genes 010 we inferred have no significant effect on choosing the host range of viruses, similar to the analysis result of the sequence above. By contrast, the rest of the four ANK genes were more complex and mutable. For example, the uncertain genes of some virus strains such as the ANK genes138 of vaccine strains of both GTPV-Gor and YM, don’t even have ANK repeats and F-box like domains, while the ANK repeats numbers of the same ANK genes from different virus strains also have larger differences, e.g., the isolated strain of GTPV-SS contain seven repeat domains in ANK genes138 but no yet presenting in another several virus strains of capripoxvirus.

Moreover, the ANK genes 145 of almost all SPPV have the F-box like domains but no present in GTPV, confirming the above analysis result that 100 bp was deleted from the ANK genes 145 of GTPV. For the five ANK genes from the same virus strains, vaccine strains of capripoxvirus contain fewer F-box domains than those of isolated strains of capripoxvirus, consistent with the analysis results of amino acids. Overall, in five ANK genes of capripoxvirus especially ANK genes 138,140,141.2 and 145 in determining host range, need the combining of at least two genes, any individual genes can’t be enough effectiveness for the virus choose host species, consistent with the analysis result of gene sequences, just as Tulman et al. [26] by analyzing SPPV strain NK, found that simultaneous deletion of ORF 138 and 141.2 of capripoxvirus would significantly affect the virus’s preferendum or virulence. Besides, further analyzing also found that so far, the frameshift of the attenuating vaccines of capripoxvirus, whether SPPV vaccine or GTPV vaccine strains only present the ANK genes with F-box domains, while the relating literature proposed after ubiquitination of proteins, F-box architecture can exploits the ANK repeats to dictate targeting specificity [22], further revealed the significance of F-box domains and the ANK repeat combining of capripoxvirus in attenuating virulence or change host. More importantly, the present or deletion of F-box like domains of ANK gene 145 in the process of virus choose host can play the key roles, from these above data again indicated that ANK genes in function do have obvious comprehensive roles.

Another researcher found that there is a common feature for the majority of vaccines strains of capripoxvirus that commonly present frameshift mutations of single ANK genes and uncertain number kelch family protein genes as virulence factors (ORF 019,137 and 144) such as SPPV strains of Turkey (MN072631.1), Saudi Arabia (MN072627.1) NISKHI (AY077834), and GTPV strain of Gorgan (KX576657.1) and G20-LKV (AY077836.1) [6, 14], can achieve the aim to immunize goats or sheep. Whether this is the case requires a large number of experiments to prove in the future.

The experiment mainly aimed to trace the source of the capripoxvirus outbreak in 2010 in Xinjiang, and analyze the evolutionary relationship of ANK gene-family as viral host range factors, with effect mechanism of host range genes, and to establish foundations for further study of cross-species transmission of capripoxvirus.

## Conclusions

(1) The five ANK genes’ sequences of three SPPV and two GTPV isolates strains from Xinjiang province in 2010 were closer to India’s virus strains, respectively, so the outbreak of capripox in Xinjiang in 2010 may have originated in India. (2) There was happened cross-species infection in this outbreak by a comparative analysis of the five genes’ sequences of isolates strain GTPV-SS with others. (3). As host range factors, the ANK proteins play a species-specific role by combined all of five ANK proteins according to analyze the number of code-shift mutations of their amino acid sequence of the isolated strains and vaccine strains of capripoxvirus.

## Data Availability

These sequencing data used in the current experiment were available by contacting the corresponding author.

## Abbreviations

ANK gene: Ankyrin gene
ORF: Opening read frame
OIE: Office of International Epizootic
*E. coli* DH5α: *Escherichia coli* DH5α
GTPV: Goatpox virus
SPPV: Sheeppox virus
LSDV: Lumpy skin disease virus
