# Correction to: Comparative analysis of ankyrin (ANK) genes of five capripoxviruses isolate strains from Xinjiang province in China

**DOI:** 10.1186/s12985-021-01534-y

**Published:** 2021-06-01

**Authors:** Chuanchuan He, Jianjun Tong, Xueping Zhang, Milikaimu Tuohetiniyazi, Yu Zhang, Youwen Li

**Affiliations:** 1 Key Laboratory of Tarim Livestock Science Technology, Alar, 843300 Xinjiang China; 2 College of Animal Science, Tarim University, Alar, 843300 Xinjiang China; 3 College of Life Science, Tarim University, Alar, 843300 Xinjiang China

## Correction to: Virology Journal (2020) 17:133 10.1186/s12985-020-01407-w

Following the original article [1], it has been raised that tables 5, 6, and 7 in this paper bear many similarities to the tables from [2] (cited as reference 10 in [1]). We want to clarify this point in this correction.

We acknowledge that we followed the same table format used in [2], but the data came from our experiments and were different from data previously reported in [2].

In Table 5 (ANK gene 138), there are eight different nucleotide (nt) mutations, such as nt 1293, 1543, 1896, 1897–1905, and deletion mutations of nt 1895 and 1080–1082. The amino acid (aa) 631 and its following 3 aa were deleted in our sheeppox virus.

In Table 6 (ANK gene 140), there are an insertion mutation of nt105 and a deletion mutation of nt28, and One aa is missing (10) in our sheeppox virus.

In Table 7 (ANK gene 141.2), there are two more nt mutations than that of Ref.10, such as nt 1253 and 552, and 1 aa was inserted (418) in our sheeppox virus.

We also want to clarify why the results found are similar in our paper [1] and [2]. Firstly, the two papers employed similar methods to investigate the same genes (138, 140, 141.2) of capripoxvirus strains from their respectively different regions: our 6 strains were obtained from Xinjiang, China, while those analyzed in [2] were from India.

Secondly, all of the virus strains in the two papers may come from the same progenitor. China and India are neighboring countries, and Xinjiang shares a direct border with India. According to the ecological evolution of the virus in the geographical environment, it is reasonable to think that the virus found in these two regions comes from the same or similar progenitor, and that they are highly homologous.

Moreover, particularly for capripoxvirus, the genomes from different species are very similar, and the similarity is higher than 96%, and it was even more conserved for the ANK gene.

Full sequencing data (raw data and assembling sequences) are included here as Additional file 1.

## Supplementary Information

**Additional file 1**. Sequencing results of 3 ANK genes of 6 capripoxviruses isolates in Xinjiang.

## References

[1] He C, Tong J, Zhang X (2020). Comparative analysis of ankyrin (ANK) genes of five capripoxviruses isolate strains from Xinjiang province in China. Virol J.

[2] Chaple AR (2019). Genetic studies of terminal regions of vaccine and field isolates of capripoxviruses. Infect Genet Evol.

